# The process of coping with stress by Taiwanese medical interns: a qualitative study

**DOI:** 10.1186/s12909-016-0534-3

**Published:** 2016-01-12

**Authors:** Chun-Hao Liu, Woung-Ru Tang, Wei-Hung Weng, Yu-Hsuan Lin, Ching-Yen Chen

**Affiliations:** School of Medicine, College of Medicine, Chang Gung University, 259 Wen-Hwa 1st Road, Kwei-Shan, Taoyuan Taiwan; Department of Psychiatry, Chang Gung Memorial Hospital at Linkou, NO.5, Fusing Street, Kwei-Shan, Taoyuan Taiwan; Graduate Institute of Nursing, College of Medicine, Chang Gung University, 259 Wen-Hwa 1st Road, Kwei-Shan, Taoyuan Taiwan; Institute of Brain Science, National Yang-Ming University, No. 155, Sec. 2, Li-Nong St., Beitou, Taipei 11221 Taiwan; Department of Psychiatry, Chang Gung Memorial Hospital at Keelung, 222, Maijin Road, Keelung, Taiwan

**Keywords:** Medical education, Intern, Stress, Coping strategy

## Abstract

**Background:**

Internship, the transition period from medical student to junior doctor, is highly stressful for interns in the West; however, little is known about the experience of interns in coping with stress in Taiwan. This study aimed to develop a model for coping with stress among Taiwanese interns and to examine the relationship between stress and learning outcomes.

**Methods:**

For this qualitative study, we used grounded theory methodology with theoretical sampling. We collected data through in-depth interviews and participant observations. We employed the constant comparative method to analyse the data until data saturation was achieved.

**Results:**

The study population was 124 medical interns in a teaching hospital in northern Taiwan; 21 interns (12 males) participated. Data analysis revealed that the interns encountered stressors (such as sense of responsibility, coping with uncertainty, and interpersonal relationships) resulting from their role transition from observer to practitioner. The participants used self-directed learning and avoidance as strategies to deal with their stress.

**Conclusions:**

A self-directed learning strategy can be beneficial for an intern’s motivation to learn as well as for patient welfare. However, avoiding stressors can result in less motivation to learn and hinder the quality of care. Understanding how interns experience and cope with stress and its related outcomes can help medical educators and policy makers improve the quality of medical education by encouraging interns’ self-directed learning strategy and discouraging the avoidance of stressors.

## Background

Internship is the transition period from being a medical student to becoming a junior doctor, and it typically entails considerable stress [1]. Before their internship, medical students tend to be observers on clinical rotations and attend classroom lectures [1, 2]; interns, however, bear responsibility for primary care and have to work with multidisciplinary teams [3]. This increased responsibility for patient care and the long working hours create stress for interns [4, 5]. In Taiwan, the system of medical education was adopted from Japan, which occupied Taiwan from 1895 to 1945; the current system involves a 7-year programme, which includes 2 years of clinical clerkship and a 1-year rotating internship [6]. During the internship year, medical students assume responsibility for primary care with inpatient services and are frequently on call for night duty [7].

Stress in medical students has been well studied [8–10]. Interns have been found to have higher levels of emotional distress than attending physicians and residents [11]. Rosel found that stress among medical students increased compared with the level before they entered medical school; such stress may impair the quality of their primary care [12]. Poorly managed stress experiences could lead to burnout in junior doctors and result in diminished patient care [13]. To achieve a better transition from medical student to junior doctor in Taiwan, a new policy, involving changing the internship to a 2-year postgraduate year, is currently under discussion. Understanding the stress experiences and coping strategies of interns could help medical educators manage stress among interns, improve their learning outcome, and increase patient safety. However, few qualitative studies have examined the stress experiences and coping strategies of interns. This study aimed to explore the process of coping with stress among medical interns in Taiwan as an important reference for future policy in medical education.

## Methods

### Design, setting, and participants

Grounded theory methodology is a qualitative research approach developed by Glaser and Strauss in 1967 for answering process-related research questions with a focus on theory development [14]. The present study used grounded theory methodology to examine the stressful experiences of medical interns and was conducted at the Linkou branch of Chang Gung Memorial Hospital, a medical centre in northern Taiwan. The potential participants were 124 interns at the medical centre; 38 were recruited by posting an announcement on the centre’s official bulletin board, inviting participation. We employed theoretical sampling to guide the recruitment of participants who could help generate theory [14, 15]. We therefore selected participants who varied by sex and the sequence of training rotation for in-depth interviews. We interviewed 21 participants (12 male, nine female; age 24–30 years) during their final month of internship. All the participants were interviewed by the first author until data saturation (theoretical sufficiency) was achieved. Data saturation refers to the point where no new information is being provided to offer further insight into the data [16].

### Ethics

The study was approved by the institutional review board at Chang Gung Memorial Hospital. Participants signed an informed consent form and were notified of their right to withdraw from the study at any time. They were also informed that they could omit any questions they did not wish to answer without consequences for their studies or work.

### Data collection

The interviewer was a medical intern experienced in qualitative research working under the supervision of a senior researcher. We conducted participant observation along with semi-structured in-depth interviews (Table 1). Each interview began with a broad question: “Please describe your experience during your internship.” New questions developed during the course of the interviews and became increasingly focused. With the participants’ permission, we audio recorded all the interviews, and they were transcribed verbatim by the first author, who was familiar with medical terminology and knew the participants personally.

**Table 1:** The semi-structured interview guide

Questions	
(1) Please describe your experience during the internship	
(2) What is the difference between internship and clerkship	
(3) Please share your experience of stress during internship	
(4) Please talk about the night duty	
(5) Is there any influence of your emotion in the internship?	
(6) How about your sleep in the internship?	

### Data analysis

In line with grounded theory methodology, the process of sampling, data collection, and data analysis occurred in a cyclical process [17]. After finishing the first interview, the investigator analysed the data, adjusted the semi-structured interview guide, and invited the next participant to enhance the information obtained from the subsequent interview.

**Fig. 1:**
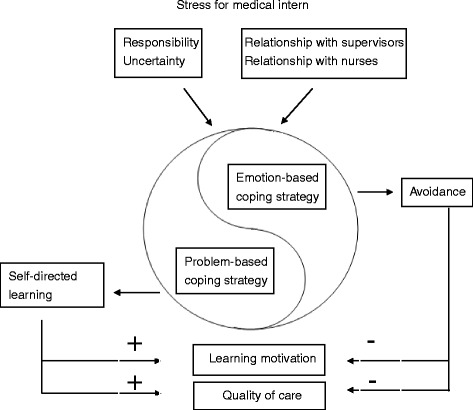
Process of coping with stress during internship. A problem-based strategy, such as self-directed learning, leads to greater learning motivation and better quality of care. By contrast, an emotion-based coping strategy, such as avoidance, leads to less learning motivation and poorer quality of care

We adopted a qualitative constant comparison method to analyse the interview data [18]. First, the investigator transcribed the interview recording verbatim within 48 h of completing each interview. Second, we coded the raw data line by line and grouped together similar codes to form a category. Third, as the study progressed, we performed continuous coding to connect categories with one another to establish their relationships and identify higher themes [19]. During the constant comparison process, the investigator continuously looked for similarities and differences in the data between participants’ narratives and among categories that developed during the process [15, 17]. Because the aim of grounded theory methodology is to generate theory that emerges from qualitative data analysis, we developed a conceptual model for this study on the process of coping with stress during internship (Fig. 1).

## Results

The stressors of the medical interns, including sense of responsibility, coping with uncertainty, and interpersonal relationships, were caused by the role transition from observer to practitioner (Fig. 1). Almost all the participants used both self-directed learning and avoidance as strategies to cope with stress.

### Stress factors related to role transition

During the transition period from observer to practitioner, the interns experienced stress from responsibility and uncertainty as well as new interpersonal relationships.

#### Responsibility

In contrast to clerkship, the participants during internship began dealing with actual patients instead of simply being observers. They began to practice their knowledge and skills in the real world and perceived how those qualities were directly related to the patients’ welfare. Participant M01 stated with respect to stress, “Interns have responsibility. You need to deal with clinical conditions… As clerks, we were just observers, but interns should know what to do; otherwise, the patient’s condition may deteriorate.” The experience of role changing was exciting but also stressful, as participant F16 indicated: “After having been a clerk, I suddenly found as an intern that I was a member of the medical team. I had the right to make decisions, but that also caused stress. At the beginning it was exciting, but then the possibility of making mistakes in my practice was stressful.”

#### Uncertainty

Another difference between clerks and interns is that the latter face the uncertainty of clinical conditions, especially when on night duty, and this was regarded as significant stress. Participant F02 felt stress owing to “uncertainty about my duty area. You never know when a patient will be short of breath, or a patient may suddenly develop serious problems while you are examining them.” As a junior doctor with limited clinical experience, the most stressful time is dealing with the uncertainty over whether to call for help. Participant M20 described this as recognizing the “patient on the edge of a cliff”: “That stress came from some clinical condition I didn’t fully understand… I wasn’t able to recognize the patient on the edge of a cliff. I didn’t know which patients were suddenly going to drop.”

#### Relationship with supervisors

As new members of a medical team, interns have to work with their supervisors and nurses. This change may cause stress. Interns were eager to show their ability to their supervisors, as described by participant M06, “I was worried about disappointing my supervisors. They never said anything directly, but I could feel some disappointment in their facial expressions and actions. They wanted me to fit in, but I failed.” Because of their personal expectations, interns may hesitate to call for help, which can further increase their stress in dealing with some clinical uncertainty. Participant M15 expressed this anxiety: “Although I could consult my supervisor whenever necessary, sometimes I didn’t want him to see me as incompetent… I had to live up to my personal expectations.”

#### Relationship with nurses

The relationship with nurses can also be stressful. The interns felt that they had to live up to the expectations of nurses, as participant M15 said, “I was under stress from my supervisor, my co-workers, and the nursing staff. I was afraid that I could not meet their expectations.” The interns had to establish new relationships with their co-workers. If interns have an argument with nurses, that becomes a source of stress in the workplace. Participant F07 explained this: “I seldom express my emotion in the workplace, but one time I expressed my anger directly in front of other people… I was under stress and felt that my clinical performance would become impaired because of a conflict I had with the nurses. We still have to work together.”

### Different coping strategies and their outcomes

Participants used self-learning and avoidance as their strategies to cope with their stress. Different coping strategies can lead to different outcomes in interns’ motivation with respect to learning and the quality of the care they administer.

#### Self-directed learning beneficial to motivation and patient welfare

Taking care of patients is stressful; however, the stress can have positive effects through the use of a problem-based coping strategy, such as self-directed learning. Participant F16 explained the relationship between stress and learning: “When taking care of a patient, I am under some stress regarding my ability to help them. But at the same time, I want to transform my textbook knowledge into treatment for the patient.” Participant M15 commented on using a problem-based strategy to deal with stress caused by a patient asking a clinical question: “I’m the one dealing with the patient, so I have to identify the problems and solve them. I need to read more textbooks to improve myself so that I can handle clinical problems.”

By means of a problem-based strategy, stress can transformed to the motivation to learn and solve clinical problems. Interns can thereby gain a sense of achievement through having participated in medical practice and obtaining positive feedback for their motivation. Participant M11 made the following comment about being an intern: “It means you can participate in the process of solving a patient’s complaints and follow the progress of their disease. That’s the major difference between being an intern and a clerk. It may also make you want to learn more.”

In addition to increased motivation, self-directed learning can improve patients’ welfare by increasing the quality of their care. Interns tend to apply the knowledge they gained when solving patients’ problems in their clinical practice. Participant F02 had experience of transforming stress into motivation and improving the quality of care: “I set a higher standard for myself, which leads to stress. For example, when dealing with some difficult clinical problems, because of my personal expectations [in caring for patients] . . . I pushed myself to search databases for evidence-based medical findings so that I could give patients a more complete answer.”

#### Avoidance leading to negative learning outcomes reduces quality of care

If stress proved overwhelming or interns were unable to make a timely response to their supervisors, the interns were able to use an emotion-based strategy, such as avoidance. By adopting an emotion-based strategy, participants tried to avoid the stress instead of attempting to deal with it. Participant M09 said, “The stress came from my being unable to deal with a situation… I was in a rush and didn’t know what I should do. I became afraid… I felt like running away.” Participant F07 echoed that view: “When I was a clerk, I viewed clinical problems as an opportunity to learn, and I thought I might be able to do something to help patients. But during my internship, I just wanted to escape and hoped I might not have anything further to do with the situation.” By using an emotion-base strategy, such as avoidance, the participant allowed stress to reduce the motivation to learn: “When I was overwhelmed [by stress], I had no interest in learning about it [the clinical problem] and received no benefit from the clinical work.”

Moreover, using an emotion-based strategy may also reduce the quality of care because of irritability, impatience, and avoiding the need to solve problems. Participant F18 made the following comment: “Night duty was stressful. When I was in a good mood, I could have detailed consultations with patients, but I couldn’t do that when I was stressed out.” Participant M20 summarized the experience with an emotion-based strategy and its negative effect: “I’m more irritable and easily angered than when I was a clerk. Then, I used to spend more time with patients, dealing with the clinical problems. But now, I’m more irritable and just want to finish the job as quickly as possible.”

We were able to observe both an emotion-based strategy and a problem-based strategy in the same participant during observations and interviews. We assume that these two strategies may be used together and that the outcome depends on their respective proportions.

## Discussion

To the best of our knowledge, this is the first study to employ a grounded theory approach to explore the experiences of medical interns with respect to stress. We also believe it to be the first study to attempt to establish a model of the process interns use to cope with that stress. Most of our participants indicated that one of the major sources of stress was being unprepared for responsibility; this was previously reported for newly qualified doctors in the United Kingdom [1, 20]. Alexander and Haldane also identified the stressful nature of increased responsibility during the transition period [4]: unlike clerks, interns had to deal with uncertainty regarding a patient’s condition, and the interns viewed that as a cause of stress. This result is similar to that of a British study, which found that some newly qualified doctors viewed uncertainty as stressful [1].

Adapting to their new role and familiarizing themselves with novel interpersonal relationships with other members of a medical team can also cause stress for interns. In the present study, Taiwanese interns identified the interpersonal interactions during their role shift as a source of stress; this has also been reported for interns in Australia and the United States [21, 22]. Timely support provided by medical educators, however, can turn stress into the motivation to learn. Dornan and colleagues found supportive participation to be the core condition for workplace learning [23]. Real-time support from senior colleagues, such as a full time medical educator, can be vital in reducing stress for interns. To achieve better preparation for stress, medical educators should remind interns that their uncertainties were once shared by their clinical tutors; educators should also stress the importance of interpersonal relationships based on understanding and respect.

In this study, we found that depending on the coping strategy used, stress could have a positive or negative effect on interns’ learning motivation and quality of care. A previous study showed that emotional symptoms were significant in a highly stressful environment and may lower the standard of care [24]. Stress can cause suboptimal care and a decline in empathy [10, 13, 25]. Regular monitoring of interns’ negative emotions and immediate interventions is important not only for the welfare of the interns themselves but also for that of their patients.

By contrast, we found that a more problem-based coping strategy was an important factor for improved learning and clinical practice. A qualitative study in Ireland suggested that increasing interns’ responsibilities could motivate learning [26]. Another study also determined that learning from real patients made clinical learning easier, meaningful, and focused for interns [27]. As suggested by the conceptual model of coping reserve, Dunn and colleagues demonstrated how interns used problem-based coping to perceive a positive experience [28]. Their model indicated that positive and negative factors during medical training could lead to resilience or burnout; they also found that different outcomes were determined by personal characteristics. We found the strategy adopted by interns to be the key factor in the different outcomes; however, it is necessary to examine further how interns decided to choose each strategy and how that is related to their underlying personality.

We observed that problem-based and emotion-based strategies can exist concurrently and in different proportions. This finding may explain previous contradictive results about coping strategies. One study in the United States revealed that 4th-year medical students used a more confrontational strategy, such as increasing aggressiveness, than did 1st-year students in coping with interpersonal stress [29]. Conversely, another US study found problem solving to be the most frequently used coping strategy by medical graduates [30]. A study of US medical students determined that a problem-based strategy could reduce emotional problems caused by stress [31]. That may be because a more problem-based strategy can overcome an emotion-based strategy, thereby reducing the negative consequences of stress. Clinical teachers should remind current and future interns about the importance of self-directed learning as a strategy to relieve stress and the negative effect of using an emotion-based strategy for that purpose.

One strength of this study was that the participants were interviewed by a medical intern who participated in all internship training courses; that intern made observations in the field for 1 year and could understand and sympathize with the participants’ experiences. There are some limitations to this study. First, this was an exploratory qualitative study with a relatively small sample size. The results of this study need to be confirmed by further quantitative studies with larger sample sizes. Second, as noted, the interviewer was a medical intern who participated in all training courses: although this may have increased the credibility of the interviews, it could also have resulted in a self-selection bias in the interviews when participants had to respond candidly to a peer.

## Conclusions

This study explored the stressful experiences of medical interns and their coping strategies during the role transition from observer to practitioner. A problem-based strategy, such as self-directed learning, can turn stress into positive feedback and provide benefits in terms of motivation and quality of care. By contrast, an emotion-based strategy may cause fatigue and emotional distress. To avoid stress, a number of interns sacrificed their motivation and the quality of care. This study developed a model to examine how interns use different strategies to cope with stress and the impact of stress on their motivation to learn and quality of care.
